# Exploring Long Noncoding RNAs in Glioblastoma: Regulatory Mechanisms and Clinical Potentials

**DOI:** 10.1155/2018/2895958

**Published:** 2018-07-12

**Authors:** Tao Zeng, Lei Li, Yan Zhou, Liang Gao

**Affiliations:** ^1^Department of Neurosurgery, Shanghai Tenth People's Hospital, Tongji University School of Medicine, No. 301 Middle Yanchang Road, Shanghai 200072, China; ^2^Medical Research Institute, College of Life Sciences, Wuhan University, Wuhan 430071, China

## Abstract

Gliomas are primary brain tumors presumably derived from glial cells. The WHO grade IV glioblastoma (GBM), characterized by rapid cell proliferation, easily recrudescent, high morbidity, and mortality, is the most common, devastating, and lethal gliomas. Molecular mechanisms underlying the pathogenesis and progression of GBMs with potential diagnostic and therapeutic value have been explored industriously. With the advent of high-throughput technologies, numerous long noncoding RNAs (lncRNAs) aberrantly expressed in GBMs were discovered recently, some of them probably involved in GBM initiation, malignant progression, relapse and resistant to therapy, or showing diagnostic and prognostic value. In this review, we summarized the profile of lncRNAs that has been extensively investigated in glioma research, with a focus on their regulatory mechanisms. Then, their diagnostic, prognostic, and therapeutic implications were also discussed.

## 1. Introduction

The WHO grade IV glioblastoma (GBM), characterized by high recurrence rate, high morbidity, and mortality, is the most common, aggressive, and deadly primary intracranial tumor in adults, reflecting the urgent need to develop new diagnostic and therapeutic targets for this devastating disease [1–3]. The standard therapy for GBM is the combination of maximal surgical tumor resection, radiotherapy, and chemotherapy [1, 4]. However, the average life expectancy for GBM patients is only approximately 15 months after initial diagnosis even though optimal treatment has been received [5, 6]. GBM malignancy and poor prognosis are closely correlated with the deregulation of signaling pathways controlling tumor cell proliferation, resistance to apoptosis, invasion, angiogenesis, and immune evasion [6, 7]. Thus, investigations revealing essential molecular mechanisms governing these features of glioblastoma with potential diagnostic and therapeutic value have drawn remarkable attention [8]. With the advent of high-throughput technologies, a wide variety of noncoding RNAs, including microRNAs and long noncoding RNAs (lncRNAs), has been identified in glioma tissues and cell lines, some of which show strong functional indications [9]. Interestingly, the so-called glioma stem cells (GSCs), which are believed to be responsible for GBM initiation, therapy-resistant, and relapse, carry diverse molecular and genetic changes, including aberrant lncRNA expression [10, 11]. Additionally, the competitive regulatory network formed among lncRNAs, protein-coding transcripts, and microRNAs seems to play a crucial regulatory role in the proliferation, metastasis, and resistance to apoptosis of GSCs [12–14]. Although researches of lncRNAs in GBMs are still in infancy, exploring their roles and mechanisms would not only deepen our understandings on molecular features of GBMs but also open new windows for unveiling novel diagnostic and therapeutic targets [15]. Here, we summarize recent progress regarding GBM-associated lncRNAs that have been under intensive investigations, with emphases on their regulatory mechanisms and clinical relevance.

## 2. Overview of lncRNAs

LncRNAs, a class of RNAs greater than 200 nucleotides (nt) without significant protein-coding capacity, were initially documented in the epigenetic regulation of X chromosome inactivation during embryogenesis [16, 17]. Occasionally, functional short peptides can be derived from lncRNAs [18, 19]. Until now, the NONCODE database has annotated 87,774 and 96,308 lncRNA genes in the mouse and human genome, respectively, which are far more than protein-coding genes (PCGs) [20]. LncRNA genes are largely categorized according to their locations and transcription orientations relative to the closest protein-coding genes. Thus, they can be antisense (partially or fully overlapped with PCGs), sense, divergent, convergent, intronic, and intergenic (no PCGs within a 5-kilobase range) [21]. Compared to protein-coding mRNAs, lncRNAs transcribed from intergenic regions (lincRNAs) are less spliced and largely nonpolyadenylated and are mostly attached to chromatin [22]. LncRNAs are usually expressed at lower levels than protein-coding mRNAs and display more cell type- and tissue-specific expression patterns. Notably, about 40% of lncRNAs are mainly expressed in the brain, reflecting the cellular and functional complexity of the brain [23]. Furthermore, lncRNA expression is spatiotemporally regulated during neural development [24–27] and upon neural activity [28, 29]. Since many lncRNAs are capable of forming a complex with DNA, RNA, and proteins dynamically, they regulate gene expression at multiple levels including chromatin remodeling, histone and DNA modification, and the process of transcription, as well as RNA splicing, transport, and stability [6, 30–33]. Particularly, lncRNAs could play *cis*-regulatory roles to positively or negatively control the transcription of neighboring PCGs [34]. Although functions of most lincRNAs have yet to be unveiled, some have been found to facilitate the chromatin structure and histone modifications, to act as a coactivator or corepressor in the nucleus [35, 36] or to modulate signal transduction in the cytosol [37]. In addition, some lncRNAs bear complementary sites (also known as miRNA response elements, MRE) for microRNAs (miRNAs). miRNAs have been widely recognized to be involved in almost every facet in the development and malignant progression of gliomas, including the maintenance of the stemness of GSCs, invasiveness, angiogenesis, epigenetic regulation, and signaling pathways [38–42]. The so-called competitive endogenous RNAs (ceRNAs) function as molecular “sponges” for miRNAs via their MREs, thus derepressing the target genes of the respective miRNAs [43–45]. Circular RNAs or transcripts of pseudogenes might also behave as ceRNAs. Some studies further indicated that a few lncRNAs could modulate gene expression and/or cell signaling at multiple molecular levels simultaneously [46, 47]. Given the broad involvement of lncRNAs in cellular events, it is not surprising to reveal that a number of lncRNAs play pivotal roles in embryogenesis, tissue homeostasis, and the development and progression of various diseases [48–51].

## 3. Aberrant lncRNA Expression in Glioblastomas

Dysregulated expression of lncRNAs is associated with human diseases such as cardiovascular diseases, neurodegenerative disorders, and malignant tumors, which are also evident in brain tumors [52, 53]. Moreover, several studies showed that abnormal expression profiles of lncRNA in clinical glioma specimens are correlated with histological differentiation and malignancy grades, which may have clinical implications in the diagnosis and prognostication [54, 55]. A high-throughput screen study by Han et al. identified 1308 differentially expressed lncRNAs in GBMs compared to normal brain tissue. Among them, *ASLNC22381* and *ASLNC2081* were predicted to be involved in the recurrence and malignant progression of GBM by upregulating the expression of *IGF-1* (insulin-like growth factor 1) [56]. Similarly, a transcriptome comparison study identified differentially expressed lncRNAs and mRNAs between GBM and normal brain samples. Gene ontology (GO) and pathway analysis-predicted genes involved in GBM pathogenesis could be modulated by lncRNAs, such as the *HOX* cluster-associated lncRNAs [57]. In another study, Zhang et al. identified 129 differentially expressed lncRNAs in glioma tissues. Their analysis revealed that the levels of lncRNA *MEG3* were significantly downregulated in GBMs, whereas those of *HOTAIRM1* (HOX antisense intergenic RNA myeloid 1) and *CRNDE* were upregulated [58]. Importantly, the expression pattern of these lncRNAs correlates with histological classification and malignancy grades of gliomas [58]. Although mechanistic evidence remains to be explored, differentially expressed lncRNAs could be a start point for digging out novel biomarkers for diagnosis or targets for therapy [6]. Next, we will list individual lncRNAs regarding their involvements in multiple aspects of GBMs. In the past few years, researchers largely focused on a few well-documented, abundantly expressed lncRNAs that have explicit indications for their participation in development and/or in tumorigenesis, with some of which showing diagnostic and prognostic potentials (Table 1).

### 3.1. *HOTAIR* (HOX Transcript Antisense Intergenic RNA)

LncRNA *HOTAIR*, located at chromosome 12q13, was originally implicated in epigenetic silencing of genes at the *HOXD* locus and inhibits initiation of transcription by recruiting PRC2 (polycomb repressive complex 2) [59, 60]. Aberrant *HOTAIR* expression was closely related to cancer metastasis and was defined as a negative prognostic factor for patients with malignant tumor [61, 62]. *HOTAIR* expression in GBMs is significantly higher than that in normal brain tissues and low-grade gliomas and correlated with poor prognosis and glioma molecular subtype. Moreover, *HOTAIR* was an independent prognostic factor in GBM patients. A gene set enrichment analysis (GESA) revealed that *HOTAIR* expression primarily associated with genes involved in cell cycle progression. Consistently, *HOTAIR* maintains proliferation and tumorigenic potential of GBM cells [63]. *HOTAIR* regulates cell cycle progression of GBM cells probably through EZH2, the core component of the PRC2 (polycomb repressive complex 2) [64], and the Wnt/*β*-catenin pathway [65]. Reports also suggest that *HOTAIR* might behave as a competing endogenous RNA (ceRNAs) to regulate the levels of prooncogenic transcripts by buffering miRNAs [66, 67]. The mechanisms underlying elevated *HOTAIR* expression in glioma remain to be investigated, and a study in breast cancer hints at both transcription and posttranscriptional regulations [47]. In summary, these findings reveal that *HOTAIR* may enhance the development of GBM through multiple regulatory signals, and its clinical value awaits further study.

### 3.2. *CRNDE* (Colorectal Neoplasia Differentially Expressed)

LncRNA *CRNDE* is initially identified to be overrepresented in >90% of colorectal adenomas and adenocarcinomas [68, 69]. Later, *CRNDE* was also found to be highly expressed in brain cancers including GBM and astrocytomas [55, 69, 70]. Applying a microarray-mining approach, Zhang et al. reported that *CRNDE* was upregulated by 32-fold up in glioma tissues than that in nontumor brain tissues [58]. *CRNDE* overexpression promotes glioma cell growth and migration *in vitro* and tumorigenesis in a xenograft mouse model. Mechanistic studies suggested that *CRNDE* expression could be regulated by the mTOR signaling and the histone acetylation status in the promoter region [71]. *CRNDE* is enriched in the stem-like population of GBM cells and promotes tumor cell proliferation and migration and by sponging down miR-186 to derepressing the expression of *XIAP* (X-linked inhibitor of apoptosis) and *PAK7* [p21 protein- (Cdc42/Rac-) activated kinase 7], two prooncogenic molecules [72]. Similarly, *CRNDE* behaves ceRNAs for miR-384 to maintain the expression of *PIWIL4* (piwi-like RNA-mediated gene silencing 4), which promotes gliomagenesis probably by activating the STAT3 signaling [73]. Moreover, high *CRNDE* expression correlates with tumor progression and poor survival for glioma patients [55].

### 3.3. *NEAT1* (Nuclear-Enriched Abundant Transcript 1)

LncRNA *NEAT1* is crucial for the formation of paraspeckles, nuclear domains implicated in mRNA nuclear retention, and splicing [74–76]. *NEAT1* is upregulated in human GBM tissues [6] and glioma cell lines like U251 and U87 [77]. *NEAT1* expression was higher in glioma tissues than adjacent noncancerous tissues. Higher *NEAT1* expression correlated with a larger tumor size, higher WHO grade, recurrence rate, and unfavorable overall survival, supporting *NEAT1* as a potential prognostic predictor of glioma patients [78]. *NEAT1* has been implicated in gliomagenesis by promoting cell proliferation, invasion, and migration. Zhen et al. demonstrated that *NEAT1* could upregulate the expression of *c-Met* oncogene through buffering miR-449b-5p, a negative regulator of *c-Met* [79]. The latest study by Chen et al. showed *NEAT1* could be upregulated by the oncogenic EGFR pathway. Elevated *NEAT1* promotes GBM tumorigenesis by acting as a scaffold molecule to recruit the histone modification enzyme EZH2 to silence target-specific genes including *AXIN2*, *ICAT*, and *GSK3B*, thus leading to the activation of the canonical Wnt/*β*-catenin signaling. This study highlights the epigenetic role of lncRNAs in controlling the expression of tumorigenic components in GBM cells [80].

### 3.4. *XIST* (X-Inactive Specific Transcript)

LncRNA *XIST* is the major effector of X inactivation in mammals to balance gene expression between the sexes, and the *XIST* RNA is exclusively transcribed from the inactive X chromosome [16, 17]. *XIST* loss in female mice leads to a highly aggressive myeloproliferative neoplasm and myelodysplastic syndrome (mixed MPN/MDS) [81]. *XIST* has been found to be up- or downregulated in a variety of human cancers [82]. Yao et al. found *XIST* expression was upregulated in glioma tissues and GSCs and knockdown of *XIST* suppresses GSC proliferation, migration, invasion, and tumorigenic potential by upregulating miR-152 [14]. A few other studies also indicated *XIST* confers glioma cell oncogenic and chemoresistant behaviors by serving as ceRNAs to suppress actions of microRNAs [83–85]. One recent study pointed out a novel role of *XIST* in regulating a glioma microenvironment. *XIST* was found to be overrepresented in glioma endothelial cells (GECs), and *XIST* knockdown increased permeability of the brain-tumor barrier (BTB) and inhibited glioma angiogenesis, which may have beneficial effects on GBM treatment. Mechanistically, *XIST* regulates the expression of the transcription factor Forkhead Box C1 (FOXC1) and Zonula occludens 2 (ZO-2), two molecules essential for maintaining the BTB integrity, by dampening miR-137 [86].

### 3.5. *H19*

LncRNA *H19*, transcribed only from the maternally inherited (imprinted) allele, is involved in the postnatal development and tumorigenesis [87, 88]. *H19* is upregulated in glioma tissues and was negatively associated with patient survival time [89]. *H19* overexpression enhances invasion, angiogenesis, stemness, and tumorigenicity of GBM cells, whereas *H19* depletion has opposite effects [90, 91]. *H19* might exert its prooncogenic function via the embedding miR-675, which could target the expression of tumor suppressor gene *RB1* [12, 89, 92]. Similar to *XIST*, *H19* was also found to be highly expressed in glioma-associated endothelial cells (GECs). Depletion of *H19* inhibited glioma-provoked GEC proliferation, migration, and tube formation. Knockdown of *H19* upregulates miR-29a, resulting in decreased expression of VASH2, an angiogenic factor [93].

### 3.6. *SOX2OT* (*SOX2* Overlapping Transcript)

The genomic region that transcribes lncRNA *SOX2OT* contains the *SOX2* gene, one of the major pluripotency regulators, in its intronic region [94]. Similar to *SOX2*, *SOX2OT* is highly expressed in embryonic stem cells and neural precursor cells but becomes downregulated upon differentiation. Elevated *SOX2OT* expression and the concomitant *SOX2* expression were also noticed in some carcinomas with an epithelial origin, including lung, breast, and esophageal cancer [95]. The transcriptional regulation of *SOX2* by *SOX2OT* has been highlighted in development and tumorigenic scenarios [96–98]. A recent study found *SOX2OT* is essential for proliferation, migration, invasion, and tumorigenesis of GBM stem-like cells (GSCs). The results also indicated *SOX2OT* might act as ceRNAs to maintain the expression of *SOX3* by buffering miR-122 and miR-194-5p. Moreover, in GSCs, SOX3 functions as an oncogene and transactivates the expression of *SOX2OT* and *TDGF-1*, thus forming a positive feedback loop [99].

### 3.7. *MEG3* (Maternally Expressed Gene 3)

LncRNA *MEG3*, also known as *Gtl2 (gene trap locus 2)* in mice, is transcribed from the imprinted maternal allele, with multiple isoforms generated by alternative splicing [100, 101]. *MEG3* expression is prevalent in human normal tissues, while it becomes diminished in most human tumors, and overexpression of *MEG3* inhibits the growth of human cancer cells [102–104]. DNA methylation at the promoter or the intergenic differentially methylated region of *MEG3* mediates silencing of the *MEG3* gene in tumors. Ectopic *MEG3* expression significantly elevates the level of tumor suppressor protein p53 in human cancer cell lines. The increased p53 level upon *MEG3* overexpression is partly due to the downregulation of MDM2, an E3 ubiquitin ligase that targets p53 for degradation [105]. *MEG3* is significantly downregulated in GBMs and behaves as a tumor suppressor in GBM cells in a p53-dependent manner [106, 107]. A recent study also suggested *MEG3* might act as competing endogenous RNAs (ceRNAs) of miR-19a and miR-93 to inhibit GBM cell growth [108, 109]. Li et al. provided evidence that hypermethylation at the *MEG3* promoter mediated by DNMT1 controls the expression of *MEG3* and subsequent p53 activity in glioma cells. Moreover, treating glioma cells with the DNA methylation inhibitor 5-AzadC inhibited the growth and promoted apoptosis of glioma cells, and its potential application in GBM animal models remains to be investigated [110].

### 3.8. *MALAT1* (Metastasis-Associated Lung Adenocarcinoma Transcript 1)

LncRNA *MALAT1*, also known as *NEAT2 (noncoding nuclear-enriched abundant transcript 2)*, was initially demonstrated to be positively associated with metastasis and shorter survival in non-small cell carcinoma (NSCLC) patients, specifically in the early stages of lung adenocarcinoma [111]. Analogous to *NEAT1*, *MALAT1/NEAT2* is majorly enriched in the paraspeckle, a nuclear structure essential for RNA storage and splicing [112]. But its role in alternative splicing might be species-specific [113]. In most solid tumors, *MALAT1* is highly expressed and is associated with poorer clinical parameters [114]. Interestingly, studies in glioma have inconsistent results regarding roles of *MALAT1*. *MALAT1* expression was reported to be lower in glioma tissues than that in noncancerous brain tissues, and its higher expression correlates with better patient survival, suggesting *MALAT1* may serve as an independent prognostic factor and act as a tumor suppressor in glioma [115]. Accordingly, the proliferation and invasion ability of glioma cells was significantly enhanced by *MALAT1* knockdown in glioma xenograft models, whereas *MALAT1* overexpression had opposite effects [115]. *MALAT1* might exert its tumor suppressor role by inactivation of the prosurvival ERK/MAPK signaling and/or enhance the expression of FBXW7, an antiproliferation protein [116]. However, a few other studies indicated *MALAT1* has prooncogenic (tumor-promoting) roles and knockdown *MALAT1* might confer beneficiary effects on glioma treatment [117–119]. Thus, *MALAT1* could be either a positive or a negative regulator in glioma tumorigenesis depending on cellular contexts.

### 3.9. *TUG1* (Taurine-Upregulated Gene 1)

*TUG1* was originally identified as a lncRNA required for the normal development of photoreceptors in the mouse retina [120]. As a chromatin-associated lncRNA*, TUG1* can regulate gene expression by interacting with the polycomb repressive complex 2 (PRC2) [121]. *TUG1* is extensively related to human malignancies, reported having either tumor promoting or tumor suppressing functions in different types of cancers [122–124]. *TUG1* is expressed at significantly higher levels in GBM tissues than in normal brain tissues [125]. In GSCs, *TUG1* expression is transcriptionally induced by the Notch signaling, a well-known oncogenic pathway. *TUG1* maintains stemness and tumorigenic properties of GSCs by two parallel mechanisms: in the cytosol, *TUG1* sponges miR-145 to maintain the expression of stemness-associated genes including *SOX2* and *MYC*; in the nucleus, TUG1 is able to associate with the PRC2 and YY1 transcription factor to suppress differentiation [46]. This study underscores the importance of the Notch-lncRNA axis in regulating self-renewal of GSCs and proposes a rationale for targeting *TUG1* as a potent therapeutic approach to treat GBMs by eradicating GSCs. *TUG1* could maintain the expression of tumor suppressor PTEN by sponging off its negative regulator miR-26a, but the functional implication is not clear [125]. *TUG1* was also found to be highly expressed in glioma endothelial cells (GECs). Serving as ceRNAs for miR-144 in GECs and miR-299 in GSCs, *TUG1* is able to modulate blood-tumor barrier and enhance glioma-induced angiogenesis, respectively [126, 127]. Therefore, depleting *TUG1* in glioma cells might facilitate a microenvironment detrimental for tumor growth while beneficial for drug delivery.

### 3.10. *CASC2* (Cancer Susceptibility Candidate 2)

LncRNA *CASC2* is first identified to be transcribed from an allelic loss region at chromosome 10q26 in human endometrial cancer [128, 129]. Later on, *CASC2* was unveiled to be a tumor suppressor gene in endometrial, colorectal, lung, and renal cancers and gliomas, probably behaving as ceRNAs by buffering miR-21 and miR-18a, two microRNAs with oncogenic effects [9, 130–133]. The status of *CASC2'*s low expression is positively correlated with advanced tumor grades, shorter survival time, and poorer TMZ response in glioma patients [134]. *CACS2* overexpression could sensitize glioma cells to temozolomide (TMZ) cytotoxicity by upregulating PTEN protein and downregulating p-AKT protein through regulating miR-181a or by inhibiting autophagy via sponging miR-193a-5p to increase mTOR expression [135, 136].

## 4. Potentials of lncRNAs in Diagnostic or Prognostic Applications

Like many other malignant tumors, the clinical diagnosis of glioma traditionally relies on symptoms, imaging findings from CT/MRI scans, and histological properties of resected tumor tissues. Recent advancement in high-throughput technology enables both clinicians and researchers to acquire genome, epigenome, transcriptome, and proteome data of tumor bulk or individual tumor cells [137–139]. Incorporation of these data generates molecular signatures of tumors much more comprehensively than traditional understanding. For instance, transcriptome signatures could classify GBMs into molecularly distinct subgroups including proneural and mesenchymal GBMs, which correspond to clinical and histological properties [140, 141]. More importantly, the current WHO classification of tumors of the central nervous system is now defined by both histology and molecular features, the latter including IDH1/2 mutation, 1p/19q co-deletion, and histone H3 K27-mutant [142]. The inclusion of these molecular signatures is due to their explicit indications for prognosis and/or targeted therapy. Furthermore, sampling and molecular description of glioma tissues at multiloci or overtime for individual tumors could allow the understanding of the heterogeneity features of GBMs, as well as their evolving path molecularly [143–145]. The accumulation of this knowledge would eventually lead to design patient-tailored glioma therapeutics.

As mentioned earlier, high-throughput transcriptome analyses of glioma tissues/cells identified numerous highly and/or differentially expressed lncRNAs, including *MALAT1*, *HOXA11-AS*, and *CRNDE*, which correlate with histological and molecular subclassification, and/or show prognostic values [15, 55]. Zhang et al. carried out lncRNA profiling from 213 GBMs using data from The Cancer Genome Atlas (TCGA) database and identified six lncRNAs including *KIAA0495*, *MIAT*, *GAS5*, *PART1*, *PAR5*, and *MGC21881*, whose weighted expressions are closely associated with the overall survival of GBM patients. Further analysis demonstrated that the six-lncRNA signature was an independent risk factor for the prognosis of GBM patients [146]. Similarly, using consensus clustering of 1970 lncRNAs from the Rembrandt dataset, a study classified gliomas into three molecular subtypes called LncR1, LncR2, and LncR3. Moreover, LncR1 subtype was associated with the poorest overall survival rate, while the LncR3 subtype correlated with the best prognosis [147]. These are interesting attempts showing potential application of lncRNAs in diagnostic and prognostic purposes for gliomas (Table 1). The drop of cost for molecular diagnosis based on high throughput techniques would greatly accelerate profiling of lncRNAs in glioma patients. These efforts will unveil practical lncRNA signatures for glioma, including their genomic, epigenetic, and expression features, which should be comparable with or even better than current molecular signatures. Next-generation diagnostics based on the CRISPR-Cas technique, including DETECTR (DNA endonuclease-targeted CRISPR trans reporter) [148] and SHERLOCK (specific high-sensitivity enzymatic reporter unlocking) [149, 150], will facilitate easier tumor detection and categorization molecularly, for example, using nucleic acids extracted from the cerebrospinal fluid (CSF). These emerging diagnostic tools will have to be robustly compared to standard diagnostics to ensure sensitivity and specificity.

## 5. Potentials of lncRNAs in Targeted Therapy against Glioma

Dismal outcomes for GBM patients are mostly due to high heterogeneity and aggressiveness of glioma cells, immune-privileged brain environment, lack of effective treatment, and poor BBB penetration for most drugs [151]. Although aforementioned lncRNAs are suggested to regulate these aspects, most evidence regarding their roles in tumorigenesis is collected using *in vitro* cultured GBM cells and xenografted animal models. Thus, unbiased whole-genome screening followed by the genetic manipulation of lncRNAs in glioma animal models would identify lncRNAs with explicit oncogenic or tumor suppressing roles, which could pave the path for translational application [152, 153]. Moreover, efficient delivering of small interfering RNAs (siRNAs) and antisense oligonucleotides (ASOs) that target lncRNAs in glioma mass requires advances in engineering and material science [154, 155]. For instance, Katsushima et al. revealed that intravenous injection of ASOs targeting *TUG1* in combination with a drug delivery system induced glioma stem cell differentiation and repressed tumor growth efficiently [46]. Alternatively, the CRISPR/Cas9-mediated editing of genome, epigenome, or RNA would be another promising approach to tackle glioma-expressed lncRNAs [156]. Notably, CRISPR/Cas9-based transcriptional activation or suppression can enhance or inhibit lncRNA expression epigenetically without modifying genomes, which could be advantageous to traditional gene-therapy approaches [157–159]. In addition, several epigenetic modulators that regulate oncogenic lncRNAs have recently emerged as novel therapeutic targets for GBM patients [110]. For example, Pastori et al. demonstrated that the inhibition of bromodomain protein BRD4 could alleviate the expression of oncogenic lncRNA *HOTAIR* in GBM patients, exerting an antiproliferation effect by inducing cell cycle arrest in GBM cells [160, 161].

## 6. Conclusion

A large variety of lncRNAs has been identified to be associated with deregulated gene expression and imbalanced biological processes in GBMs. LncRNAs were involved in nearly all facets of GBM malignancy, including cell proliferation, stemness, angiogenesis, migration, invasion, tumor immune responses, relapse, and drug resistance. However, it remains to be determined if certain deregulated lncRNAs are core causal factors in tumorigenesis and progression of GBMs. Further, using state-of-the-art biochemical and molecular approaches, we are able to precisely delineate how lncRNAs control molecular machinery and cellular functions. This knowledge is imperative for devising targeted therapeutics. In addition, the complex glioma milieu composed of microvessels, immune cells, extracellular vesicles, cytokines, and neural transmitters is indispensable for GBM propagation and invasion [42, 162]. Particularly, the latest progress using immune checkpoint inhibitors brings hopes for previously intractable tumors including GBMs [163, 164]. Hence, future studies need to identify lncRNAs that have essential roles in regulating the fate and behavior of microenvironment components in GBMs. In summary, the current understanding of GBM lncRNAs is only the tip of an iceberg, and continuing efforts will make possible developing novel RNA-based strategy to treat such a malignant tumor and bring new hopes for patients with GBM.

## Figures and Tables

**Table 1:** A list of deregulated lncRNAs in glioma with diagnostic and prognostic perspectives.

Category	LncRNA	Biological function/phenotypes	Molecular mechanisms/targets	Survival correlation	Others	References
Overrepresented in gliomas	*HOTAIR*	Maintains proliferation and tumorigenic potential of GBM cells	Associating with PRC2; regulating Wnt/*β*-catenin signaling; ceRNAs (competing endogenous RNAs) for miR-326 and miR-148b-3p	Yes	Preferentially expressed in classical and mesenchymal glioma	[64–67]
	*CRNDE*	Promotes glioma cell growth and migration in vitro and tumorigenesis in a xenograft mouse mode	ceRNAs for miR-136-5p, miR-186, and miR-384	Yes		[55, 71–73]
	*NEAT1*	Promotes glioma pathogenesis	ceRNAs for miR-449b-5p to upregulate *c-Met*; associating with EZH2	Yes		[78–80]
	*XIST*	Confers glioma cells' oncogenic and chemoresistant behaviors; *XIST* knockdown increased permeability of the brain-tumor barrier (BTB) and inhibited glioma angiogenesis	ceRNAs for miR-152, miR-429, miR-29c, and miR-137	/		[14, 83–86]
	*H19*	Enhances invasion, angiogenesis, stemness and tumorigenicity of GBM cells; depletion of *H19* inhibited glioma-provoked proliferation, migration, and tube formation of glioma endothelial cells (GECs)	ceRNA (miR-29a); negatively regulating *RB1* expression	Yes		[12, 89, 92, 93]
	*TUG1*	Maintains stemness and tumorigenic properties of GBM stem-like cells (GSCs); modulates blood-tumor barrier; and enhances glioma-induced angiogenesis	Associating with PRC2 and YY1; ceRNAs for miR-26a, miR-144, miR-299, and miR-145	/	Intravenous administration of ASOs against *TUG1* induces GSC differentiation and suppresses tumor growth intracranially	[46, 125–127]
	*SOX2OT*	Maintains proliferation, migration, invasion, and tumorigenesis of GSCs	ceRNAs for miR-122 and miR-194-5p	/		[99]
	*UCA1*	Promote glioma cell proliferation, invasion, and migration; modulates glioblastoma-associated stromal cell-mediated glycolysis and invasion of glioma cells	ceRNAs for miR-182 and miR-122	Yes		[165–168]

Downregulated in gliomas	*MEG3*	Impairs *in vitro* glioma cell proliferation	MDM2-p53; ceRNAs for miR-19a and miR-93	/		[105–109]
	*MALAT1*	Tumor-suppressive function in glioma	Inactivating the ERK/MAPK signaling; enhancing the expression of tumor-suppressor *FBXW7*	Yes	*MALAT1* could be either a positive or a negative regulator in glioma tumorigenesis depending on cellular contexts	[115–119]
	*ROR*	Tumor-suppressive function in glioma	*ROR*'s expression is negatively correlated with the level of *KLF4*, a stem cell gene	/		[169]
	*TUSC7*	Suppresses cellular proliferation and invasion of glioma cells, accelerates cellular apoptosis, and inhibits TMZ resistance	ceRNAs for miR-23b and miR-10a	Yes		[170–172]
	*CASC2*	*CACS2* overexpression sensitizes glioma cells to TMZ	ceRNAs for miR-181a and miR-193-5p to upregulate *PTEN* and *mTOR* expression	Yes		[134–136]
